# A cross-sectional, population-based study measuring comorbidity among people living with HIV in Ontario

**DOI:** 10.1186/1471-2458-14-161

**Published:** 2014-02-13

**Authors:** Claire E Kendall, Jenna Wong, Monica Taljaard, Richard H Glazier, William Hogg, Jaime Younger, Douglas G Manuel

**Affiliations:** 1 C.T. Lamont Primary Health Care Research Centre, Bruyère Research Institute, 43 Bruyère St., Annex E., Ottawa, ON K1N 5C8, Canada; 2 Department of Family Medicine, University of Ottawa, 43 Bruyère St., Floor 3JB, Ottawa, ON K1N 5C8, Canada; 3 Department of Epidemiology, Biostatistics, and Occupational Health, McGill, 1020 Pine Ave. West, Montreal, QC, Canada; 4 Department of Epidemiology and Community Medicine, University of Ottawa, 451 Smyth Rd., Room 3105, Ottawa, ON K1H 8M5, Canada; 5 Ottawa Hospital Research Institute, 725 Parkdale Ave., Ottawa, ON, Canada; 6 Department of Family and Community Medicine, University of Toronto, 500 University Ave., Toronto, ON, Canada; 7 Institute for Clinical Evaluative Sciences, 2075 Bayview Ave., Room G1-06, Toronto, ON M4N 3M5, Canada

**Keywords:** Human immunodeficiency syndrome, Primary health care, Chronic disease, Comorbidity, Multimorbidity

## Abstract

**Background:**

As people diagnosed with HIV and receiving combination antiretroviral therapy are now living longer, they are likely to acquire chronic conditions related to normal ageing and the effects of HIV and its treatment. Comordidities for people with HIV have not previously been described from a representative population perspective.

**Methods:**

We used linked health administrative data from Ontario, Canada. We applied a validated algorithm to identify people with HIV among all residents aged 18 years or older between April 1, 1992 and March 31, 2009. We randomly selected 5 Ontario adults who were not identified with HIV for each person with HIV for comparison. Previously validated case definitions were used to identify persons with mental health disorders and any of the following physical chronic diseases: diabetes, congestive heart failure, acute myocardial infarction, stroke, hypertension, asthma, chronic obstructive lung disease, peripheral vascular disease and end-stage renal failure. We examined multimorbidity prevalence as the presence of at least two physical chronic conditions, or as combined physical-mental health multimorbidity. Direct age-sex standardized rates were calculated for both cohorts for comparison.

**Results:**

34.4% (95% confidence interval (CI) 33.6% to 35.2%) of people with HIV had at least one other physical condition. Prevalence was especially high for mental health conditions (38.6%), hypertension (14.9%) and asthma (12.7%). After accounting for age and sex differences, people with HIV had significantly higher prevalence of all chronic conditions except myocardial infarction and hypertension, as well as substantially higher multimorbidity (prevalence ratio 1.30, 95% CI 1.18 to 1.44) and combined physical-mental health multimorbidity (1.79, 95% CI 1.65 to 1.94). Prevalence of multimorbidity among people with HIV increased with age. The difference in prevalence of multimorbidity between the two cohorts was more pronounced among women.

**Conclusion:**

People living with HIV in Ontario, especially women, had higher prevalence of comorbidity and multimorbidity than the general population. Quantifying this morbidity at the population level can help inform healthcare delivery requirements for this complex population.

## Background

The accumulation of chronic conditions over the lifespan is a significant and rising burden on individuals and healthcare systems. In Canada, 33% of community dwelling individuals report having at least one of 7 common chronic conditions [1]. There is strong evidence that the management of chronic diseases is most effectively and economically provided in well-supported primary care settings [2,3]. The community-based management of these chronic conditions consumes substantial healthcare resources [4], and the bulk of this management occurs in primary care [5,6].

As people with Human immunodeficiency syndrome (HIV) on combination antiretroviral therapy (ART) are now living longer, they are likely to acquire additional chronic conditions related to normal aging as well as from the effects of HIV and its treatment [7,8]. While the literature regarding what factors contribute to the prevalence of specific conditions is evolving, it is clear that multimorbidity, the presence of several of these conditions, is increasingly the norm for people with HIV [9]. Early in the era of HIV, it was considered a deadly, acute condition, requiring a specialist focus on care [10-12]. However, with ART HIV becomes a chronic condition, and a generalist perspective may now have a more major role as comorbidities increase [13-17]. In order to determine the best way to integrate primary and specialist care [18], we require a full understanding of the complex health needs of this population.

The specific objectives of this study are to describe the prevalence of comorbidities and their multimorbidity for people living with HIV and to compare this prevalence to an age and sex adjusted general population. In Ontario, Canada, administrative data for over 13 million multiethnic residents is routinely collected at the time of care in a single-payer health system. These data can be used to measure the prevalence of chronic disease [19]. To our knowledge, this is the first study to use a population-based approach to measure the types and numbers of chronic diseases associated with HIV. As a result, this study will inform the complex healthcare needs of this population.

## Methods

### Study design

We conducted a retrospective observational study to examine the demographic and clinical characteristics of people living with HIV in Ontario compared with those in the general Ontario population. We analyzed the administrative databases held at the Institute for Clinical Evaluative Sciences (ICES) from the province of Ontario, Canada comprising data on almost 13 million individuals (2008). These data are made available to accredited researchers through a data sharing agreement with the Ontario Ministry of Health and Long-Term Care and are individually linked using an anonymous identification number in accordance with the provincial Personal Health Information Protection Act. The study was approved by the Ottawa Hospital Research Ethics Board and the Sunnybrook Health Sciences Centre Research Ethics Board.

### Study population

**Figure 1:**
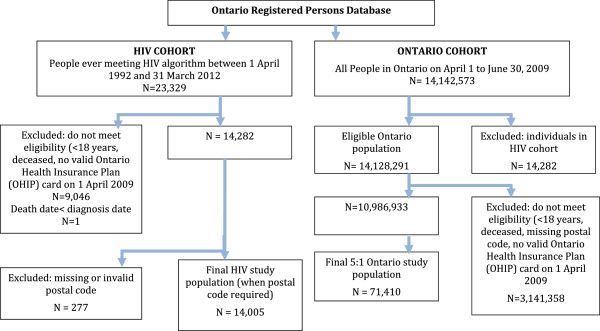
Flow diagram for study cohorts.

We identified eligible individuals from the Registered Persons Database (RPDB), an electronic registry of all Ontarians eligible for health coverage that captures patient demographic information, including age, sex, postal code and mortality data (see flow diagram Figure 1). We used data from the Ontario Health Insurance Plan (OHIP) billing claims system, which records claims for about 95% of physician services conducted in the province, to obtain an HIV cohort. We applied a previously validated algorithm to people 18 years of age and older and living in Ontario between April 1, 1992 and April 1, 2009 [20]. Briefly, this algorithm requires 3 physician claims (International Classification of Diseases, Ninth Revision (ICD-9) code for HIV infection (042, 043, 044)) over a 3-year period and has a sensitivity and specificity of 96.2% (95% CI 95.2% - 97.9%) and 99.6% (95% CI 99.1% - 99.8%), respectively for identifying people living with HIV and receiving HIV care. As a comparison, a cohort of individuals 18 years or older, living in Ontario on April 1, 2009, and not in the HIV cohort was created using a computerized random number generator in a 5:1 ratio to the HIV cohort. For both cohorts, individuals without a valid health card, age or sex value and those without valid postal codes were excluded.

### Variables

All variables for patients in the HIV and Ontario cohorts were captured using identical methods. Age, sex and postal code on April 1, 2009 were obtained from the RPDB. To broadly describe the populations, we used postal codes at the neighborhood level linked to 2006 Statistics Canada census data to assign income quintiles, marginalization quintiles and rurality scores. We used Matheson’s Canadian Marginalization Index [21] to assign marginalization quintiles for four components of marginalization (1 lowest to 5 highest): dependency, residential instability, material deprivation, and ethnic concentration and present these as a summary score. Rurality was assigned categorically into major urban areas (score 0 to 9), non-major urban areas (10 to 44), and rural areas (45 or higher) according to the Rurality Index of Ontario [22].

Using OHIP billing claims data, the Canadian Institute for Health Information (CIHI) Discharge Abstract Database, which supplies information on acute care hospitalizations and the CIHI National Ambulatory Care Reporting System, which captures information on visits to emergency departments, we applied additional previously validated algorithms in both cohorts to identify the status (i.e. presence or absence) of the following physical comorbidities on April 1, 2009: diabetes, congestive heart failure, acute myocardial infarction, hypertension, asthma, chronic obstructive lung disease, stroke, end stage renal disease and peripheral vascular disease [19,23-26]. People with mental health conditions between April 1, 2007 and April 1, 2009 were broadly captured using an algorithm previously validated to identify people receiving mental health services in the primary care setting [27].

Disease count was used to measure the prevalence of multimorbidity [28]. Multimorbidity was defined as the presence of two or more listed physical chronic conditions, and physical-mental health comorbidity was defined as a combination of mental health condition and any physical chronic health condition. In patients in the HIV cohort, this multimorbidity is in addition to HIV as their index condition.

We used the Ontario Drug Benefits claims database to identify individuals in the cohorts who were prescribed drugs paid for by the public system which covers those aged 65 and older and those receiving social assistance (Ontario Works, Ontario Disability Support Program, or the subsidized Trillium program).

### Statistical analysis

We used descriptive statistics to describe the demographic characteristics of the two cohorts. For our descriptive analyses, age was treated as both a continuous variable and a categorical variable. Sex, age group, income quintiles, rurality categories and use of Ontario Drug Benefits were described as categorical variables, and the marginalization composite score as a continuous variable. These characteristics were compared between the cohorts using two-sample t-tests for continuous variables and chi-squared tests for categorical variables. We calculated the prevalence of individual physical and mental health comorbidities, multimorbidity, and physical-mental health comorbidity in the HIV cohort, together with 95% confidence intervals (CI).

We used direct standardization to calculate age and sex standardized prevalence rates for both populations. The Canadian 1991 population was used as the reference standard [29]. We present the comparative ratio of these rates together with 95% confidence interval, calculated using the formula provided by Breslow and Day [30]. We also compare the prevalence of comorbidity and multimorbidity with age between the HIV and Ontario cohorts for both men and women. All statistical analyses were performed using SAS version 9.2 (SAS Institute, Cary, North Carolina).

## Results

Table 1 provides for descriptive purposes the demographic characteristics of people in the HIV cohort compared to the Ontario cohort. The sex and age distributions differed substantially between the HIV and Ontario populations; people with HIV were more often male (80.5% vs. 48.8%, p < 0.001) and less likely to be in the youngest (18–35 years) or oldest (>65 years) age groups (16.8% vs. 30.2% and 4.1% vs. 15.9% respectively, p < 0.001). Overall, people with HIV were poorer (lowest income quintile 31.7% vs 19.5%, p < 0.001), lived in more marginalized neighborhoods (marginalization index 3.35 (SD 0.74) vs. 2.97 (SD 0.7), p < 0.001), were more likely to live in urban settings (89.7% vs. 72.8%, p < 0.001) and were more likely to have obtained provincial drug benefits than those in the Ontario cohort (62.8% vs. 30.1%, p < 0.001).

**Table 1:** Demographic characteristics of HIV and Ontario cohorts (April 1, 2009)

		**HIV cohort**	**Ontario cohort**	**p-value**
		**n = 14,005**	**n = 71410**	
**Age (years)**	Mean (SD)	45.36 (10.77)	46.65 (17.79)	<.001
**Age group (n, %)**	18-35 years	2,355 (16.8%)	21,600 (30.2%)	<.001
	36-50 years	7,798 (55.7%)	21,895 (30.7%)	
	51-65 years	3,283 (23.4%)	16,575 (23.2%)	
	>65 years	569 (4.1%)	11,340 (15.9%)	
**Sex (n, %)**	Male	11,270 (80.5%)	34,862 (48.8%)	<.001
**Income quintile (1 lowest, 5 highest)**	Missing	131 (0.9%)	302 (0.4%)	<.001
	1	4,441 (31.7%)	13,918 (19.5%)	
	2	2,875 (20.5%)	14,224 (19.9%)	
	3	2,280 (16.3%)	14,053 (19.7%)	
	4	2,085 (14.9%)	14,554 (20.4%)	
	5	2,193 (15.7%)	14,359 (20.1%)	
**Marginalization index**	Mean (SD)	3.35 (0.74)	2.97 (0.78)	<.001
**Rurality group**	Missing	41 (0.3%)	596 (0.8%)	
	Major urban	41 (0.3%)	596 (0.8%)	
	Non-major urban	12,563 (89.7%)	52,020 (72.8%)	<.001
	Rural	1,006 (7.2%)	13,341 (18.7%)	
**ODB claim**	Yes	395 (2.8%)	5,453 (7.6%)	
		8,789 (62.8%)	21,509 (30.1%)	<.001

The prevalence of individual comorbidities and of multimorbidity among individuals in the HIV cohort are presented in Table 2. More than one-third (38.6%, 95% CI 37.8% to 39.4%) of people with HIV had a mental health condition diagnosed within the previous 2 years. One-third (34.4%, 95% CI 33.6% to 35.2%) had at least one physical chronic condition in addition to their HIV. Comorbidities with especially high prevalence included hypertension (14.9%), asthma (12.7%), diabetes (8.3%) and chronic obstructive pulmonary disease (7.9%). The prevalence of multimorbidity (at least two physical conditions) was 10.8% (95% CI 10.3% to 11.3%), and of physical-mental health comorbidity was 15.5% (95% CI 14.9% to 16.1%).

**Table 2:** Comorbidities and multimorbidity prevalence of people living with HIV in Ontario (April 1, 2009)

**Comorbidity**	**Prevalence**		
**N = 14,005**	**(n)**	**(%)**	**95% CI**
**Mental health condition**	5,401	38.6%	(37.8% to 39.4%)
**Physical conditions:**			
**Myocardial infarction**	174	1.2%	(1.1% to 1.4%)
**Congestive heart disease**	233	1.7%	(1.5% to 1.9%)
**Chronic obstructive pulmonary disease**	1,106	7.9%	(7.5% to 8.3%)
**End stage renal disease**	223	1.6%	(1.4% to 1.8%)
**Peripheral vascular disease**	72	0.5%	(0.4% to 0.6%)
**Asthma**	1,780	12.7%	(12.2% to 13.3%)
**Diabetes**	1,167	8.3%	(7.9% to 8.8%)
**Hypertension**	2,093	14.9%	(14.4% to 15.5%)
**Stroke**	173	1.2%	(1.1% to 1.4%)
**At least one physical condition**	4820	34.4%	(33.6% to 35.2%)
**Physical-mental health multimorbidity**	2,174	15.5%	(14.9% to16.1%)
**Multimorbidity (at least two physical conditions)**	1,508	10.8%	(10.3% to 11.3%)

Table 3 presents the age and sex standardized prevalence rates for the two cohorts (standardized to the 1991 Canadian population), along with the prevalence rate ratios and 95% confidence intervals. After accounting for age and sex differences, people in the HIV cohort were more likely to have at least one chronic condition than those in the Ontario cohort (prevalence ratio 1.13, 95% CI 1.07 to 1.20). People with HIV had significantly higher prevalence of all chronic conditions except myocardial infarction and hypertension, which were not significantly different from the general population. Individuals with HIV had substantially higher multimorbidity (prevalence ratio 1.30, 95% CI 1.18 to 1.44) and physical-mental health comorbidity (1.79, 95% CI 1.65 to 1.94) than the Ontario population.

**Table 3:** Comparison of comorbidities and multimorbidity prevalence between HIV and Ontario cohorts (standardized by age and sex to Canadian 1991 population)

	**HIV cohort**		**Ontario cohort**		**HIV:ON**	
	**N = 14,005**		**N = 71,410**			
	**n**	**Prevalence**	**n**	**Prevalence**	**Prevalence ratio**	**(95% CI)**
		**(%)**		**(%)**		
**Mental health condition**	5401	40.55	15935	21.99	1.84	(1.75,1.94)
**Physical conditions:**						
**Acute myocardial infarction**	174	1.19	949	1.06	1.12	(0.78,1.60)
**Congestive heart failure**	233	3.35	1428	1.49	2.26	(1.74,2.92)
**Chronic obstructive lung disease**	1106	8.33	4621	5.33	1.56	(1.39,1.76)
**End stage renal disease**	223	1.96	631	0.76	2.57	(1.92,3.44)
**Peripheral vascular disease**	72	0.63	248	0.29	2.15	(1.35,3.40)
**Asthma**	1780	15.90	8448	12.13	1.31	(1.20,1.43)
**Diabetes**	1167	9.65	6730	8.13	1.19	(1.06,1.33)
**Hypertension**	2093	19.33	16886	20.29	0.95	(0.88,1.04)
**Stroke**	173	1.55	891	1.01	1.53	(1.15,2.03)
**At least one physical condition**	4820	38.72	26907	34.23	1.13	(1.07,1.20)
**Physical-mental health multimorbidity**	2174	16.98	7443	9.49	1.79	(1.65,1.94)
**Multimorbidity (at least two physical conditions)**	1508	14.49	9417	11.13	1.30	(1.18,1.44)

**Figure 2:**
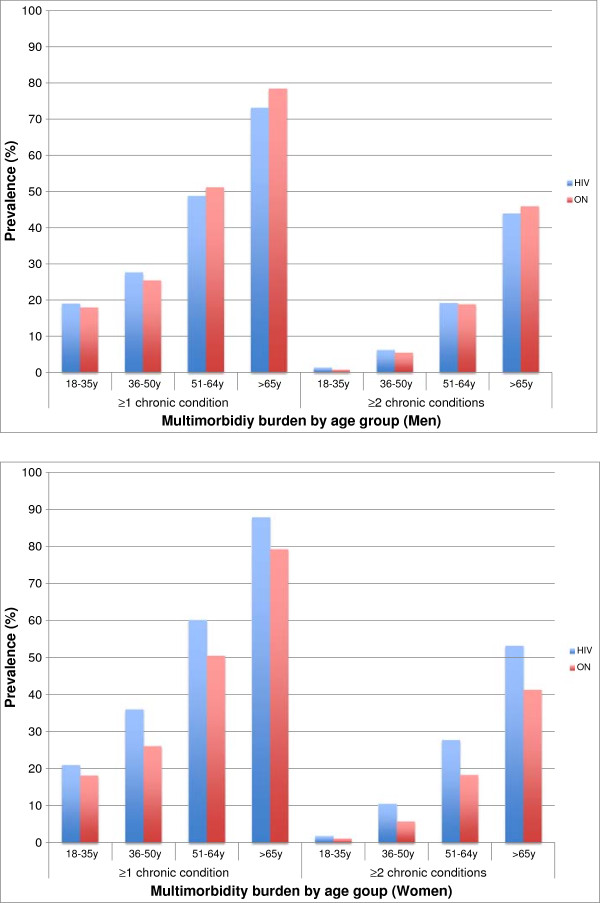
Morbidity prevalence by age group among men and women with HIV versus the Ontario general population.

Figure 2 shows the prevalence of comorbidity and multimorbidity, for men and women respectively, by age group for the HIV and Ontario cohorts. Prevalence of multimorbidity increased with age in all groups and was higher for women than men. Men in the HIV cohort only had higher prevalence of at least one chronic condition and multimorbidity than men in the Ontario cohort in the younger age groups. In contrast, multimorbidity prevalence among women with HIV was consistently higher than among Ontario women in all age groups, and this gap appeared to widen slightly with age.

## Discussion

Our study quantifies the substantial comorbidity prevalence among people living with HIV in Ontario. Mental health conditions and individual physical comorbidities were more prevalent among people with HIV than among the general population, as were multimorbidity and physical-mental health comorbidity. In addition, as is seen in the general population, our study confirms the accumulation of multiple chronic conditions with age for both men and women with HIV. Women had higher multimorbidity than men across all groups, which is consistent with the Canadian general population [1]. Furthermore, while men with HIV had slightly lower comorbidity and multimorbidity than Ontario men in most age groups, women with HIV had consistently higher comorbidity and multimorbidity prevalence than women without HIV.

To our knowledge, this is the first study to use a population-based approach to measure the types and numbers of chronic diseases associated with HIV; most studies have been conducted in clinical cohorts of people living with HIV. While the prevalence of comorbidity in people with HIV is clearly high, there have been inconsistencies regarding which conditions are more or less common in people with HIV. For instance, Butt et al. [31] found that HIV was associated with a decreased risk of diabetes, but that increasing age had a greater effect on diabetes rates in those with HIV than in the general population. Crothers et al. [32] found HIV to be an independent risk factor for COPD even after adjusting for smoking status and other risk factors. In their very large, almost exclusively male study comparing HIV positive with non HIV positive veterans, Goulet et al. [33] found that those with HIV had lower risk of hypertension, diabetes, vascular disease and psychiatric disorders, and higher rates of renal and liver disease than their HIV negative cohort. In their study of ART-experienced patients, Guaraldi et al. [8] found that those who were HIV positive had higher rates of diabetes, bone fracture and renal failure, but no difference in cardiovascular disease and hypertension. It is likely that the prevalence of individual comorbidities is a result of the complex interplay of aging, behavioral risk factors such as smoking (known to be higher among those with HIV), genetic risk factors, HIV severity, and ART history [8,9,34-36].

This study offers the unique strength of presenting the multimorbidity of people with HIV from a broad, multiethnic population of men and women from a variety of socioeconomic groups. Our findings are consistent with the literature highlighting that multimorbidity is common for people with HIV [7,8,33,34]. However, previously published prevalences of multimorbidity vary depending on the population base of the study, as clinical cohorts of people with HIV are often limited to certain demographic populations or at-risk groups. For example, our study showed that women with HIV have excess multimorbidity, which is consistent with Salter’s study of HIV positive injection drug users, but not with Guaraldi’s study of polypathology in ART-experienced people with HIV enrolled in a metabolic clinic. As men have historically represented a larger but decreasing proportion of people living with HIV, it is possible that Ontario men who were infected earlier in the epidemic were sicker and had higher mortality, resulting in a healthier cohort of older HIV-positive men within our population. However, because our population base isn’t limited by the definition of any high risk cohort and presents the clinical experience of people currently living with HIV our data is likely to be a robust estimate of the prevalence of comorbidities in the population.

In addition, previous studies have varied with respect to definitions for multimorbidity, such as specific diseases included, methods of clustering conditions, and numbers of conditions required to meet multimorbidity criteria. [5,37-40]. It is likely that broadening our comorbidity measures would have resulted in higher prevalence of multimorbidity [7,33]. Finally, in finding that almost 50% of people with HIV who have at least one comorbidity also have a mental health diagnosis, our study is the first to our knowledge to quantify the relationship between these conditions. Barnett et al. [37] found that those living in the most deprived areas had the highest prevalence of physical-mental health comorbidity at 11.0%, which is lower than the prevalence found in our HIV population, despite their higher deprivation compared to the Ontario general population.

There are several limitations to our study. First, we did not identify those who were unaware of their HIV status, estimated to be 26% of prevalent infections in Canada [41], or those not accessing health care. Furthermore, there are some settings in Ontario, most notably community health centres, that are not included in administrative data and so HIV patients in these setting where not included in the study. Community health centres are estimated to provide primary care for about 1% of the Ontario population [42], thus our findings are unlikely to be substantially affected by missing these individuals. Second, we were initially concerned that providers, especially those who have high-volume HIV practices or those who receive incentives for providing HIV care, would have preferentially identified HIV diagnosis codes for services received over other codes for chronic conditions. This potential bias would result in under ascertainment of chronic conditions in the HIV population compared to the general population. In light of this ascertainment issue, we are confident in our finding that, compared to the general Ontario population, the prevalence of almost all chronic conditions among those with HIV is higher.

## Conclusion

This population-based study quantifies the substantially higher comorbidity and multimorbidity prevalence among people living with HIV relative to the general population, and the high contribution of mental health diagnoses to these conditions. This additional burden of disease increasingly defines the care needs of this population [43,44]. Strategies for chronic disease management, including for people with HIV, must be expanded with a view to multimorbidity [18,37,43,45,46]. Particular attention should be paid to the complex care needs defined by the comorbidity burden on women with HIV. By contributing to quantifying this increasing burden with age, our results can inform policy direction around HIV health services delivery. Future work will be directed at understanding how these complex patients access the healthcare system, and how providers are meeting the healthcare needs of this population.

## Abbreviations

HIV: Human immunodeficiency syndrome; RPDB: Registered Persons Database; OHIP: Ontario Health Insurance Program; SAS: Statistical analysis system.

## Competing interests

The authors declare that they have no competing interests.

## Authors’ contributions

CK designed the study and oversaw its implementation, performed the analysis, was the primary author and approved the final version of the manuscript. JW and JY were involved in the data analysis, participated in the editing of the manuscript and approved the final version of the manuscript. DGM, MT, RG and WH contributed to the concept of the study, oversaw its implementation, helped guide the analysis and participated in the writing and approved the final version of the manuscript. All authors read and approved the final manuscript.

## Authors’ information

CK is a PhD candidate in Epidemology at the University of Ottawa. She is a Clinician Investigator and Associate Professor at the C.T. Lamont Primary Health Care Research Centre and Bruyère Research Institute and Department of Family Medicine, University of Ottawa, Canada and a research fellow at the Institute for Clinical Evaluative Sciences. She holds a Canadian Institutes for Health Research (CIHR) Fellowship in the Area of Health Services/Population Health HIV/AIDS Research. JW was an analyst at the Institute for Clinical Evaluative Sciences at the time this research was completed. JY was an analyst at the Institute for Clinical Evaluative Sciences at the time this research was completed. MT is an Assistant Professor Department of Epidemiology and Community Medicine, at the University of Ottawa, Ottawa, Ontario, Canada and a Scientist at the Ottawa Hospital Research Institute, Clinical Epidemiology Program, Ottawa, Ontario, Canada. WH is Professor and Senior Research Advisor at the Department of Family Medicine, U of O, and the C.T. Lamont Primary Health Care Research Centre and Bruyère Research Institute. He holds the research Chair in Primary Health Care with a focus on Healthy Living. RG is a Senior Scientist at the Institute for Clinical Evaluative Sciences, a Scientist in the Centre for Research on Inner City Health at St. Michael’s Hospital in Toronto, and a Professor of Family and Community Medicine at the University of Toronto and St. Michael’s Hospital. DGM is a Senior Scientist, Ottawa Hospital Research Institute, Adjunct Scientist, Institute for Clinical Evaluative Sciences, Associate Professor, University of Ottawa and University of Toronto, and Associate Scientist, C.T. Lamont Primary Health Care Research Centre and Bruyère Research Institute.

## Pre-publication history

The pre-publication history for this paper can be accessed here:

http://www.biomedcentral.com/1471-2458/14/161/prepub

## References

[B1] Broemeling A-M Watson DE Prebtani F Health Outcomes Population patterns of chronic health conditions, co-morbidity and healthcare use in Canada: implications for policy and practice Heal Q 2008 11 70 76 10.12927/hcq.2008.19859 18536538

[B2] Rothman AA Wagner EH Future of primary care chronic illness management: what is the role of primary care? Ann Int Med 2003 138 256 262 10.7326/0003-4819-138-3-200302040-00034 12558376

[B3] Dahrouge S Devline RA Hogg B Russell G Coyle D Fergusson D The economic impact of improvements in primary healthcare performance 2012 Ottawa: Canadian Health Services Research Foundation

[B4] Terner M Reason B McKeag AM Tipper B Webster G Chronic conditions more than age drive health system use in Canadian seniors Heal Q 2011 14 19 22 10.12927/hcq.2011.22485 21841372

[B5] Fortin M Hudon C Haggerty JL van den Akker M Almirall J Prevalence estimates of multimorbidity: a comparative study of two sources BMC Heal Serv Res 2010 10 111 10.1186/1472-6963-10-111 PMC2907759 20459621

[B6] Muggah E Graves E Bennett C Manuel DG The impact of multiple chronic diseases on ambulatory care use; a population based study in Ontario, Canada BMC Heal Serv Res 2012 12 452 10.1186/1472-6963-12-452 PMC3532841 23217125

[B7] Hasse B Ledergerber B Furrer H Battegay M Hirschel B Cavassini M Bertisch B Bernasconi E Weber R Swiss HIVCS Morbidity and aging in HIV-infected persons: the Swiss HIV cohort study Clin Infect Dis 2011 53 1130 1139 10.1093/cid/cir626 21998280

[B8] Guaraldi G Orlando G Zona S Menozzi M Carli F Garlassi E Berti A Rossi E Roverato A Palella F Premature age-related comorbidities among HIV-infected persons compared with the general population Clin Infect Dis 2011 53 1120 1126 10.1093/cid/cir627 21998278

[B9] Justice AC HIV and aging: time for a new paradigm Curr HIV/AIDS Rep 2010 7 69 76 10.1007/s11904-010-0041-9 20425560

[B10] Rackal JM Tynan AM Handford CD Rzeznikiewiz D Agha A Glazier RH Provider training and experience for people living with HIV/AIDS Cochrane Database Syst Rev 2011 15 6 Art. No: CD003938 doi:10.1002/14651858.CD003938.pub2 10.1002/14651858.CD003938.pub2 21678344

[B11] Handford CD Rackal JM Tynan A-M Rzeznikiewiz D Glazier RH The association of hospital, clinic and provider volume with HIV/AIDS care and mortality: systematic review and meta-analysis AIDS Care 2011 24 267 282 22007914 10.1080/09540121.2011.608419

[B12] Handford C Am T Jm R Glazier R Setting and organization of care for persons living with HIV / AIDS Cochrane Database Syst Rev 2009 3 Article No: CD004348 doi:10.1002/14651858.CD004348.pub2 10.1002/14651858.CD004348.pub2 PMC8406550 16856042

[B13] Fultz SL Goulet JL Weissman S Rimland D Leaf D Gibert C Rodriguez-Barradas MC Justice AC Differences between infectious diseases-certified physicians and general medicine-certified physicians in the level of comfort with providing primary care to patients Clin Infect Dis 2005 41 738 743 10.1086/432621 16080098

[B14] Duffus WA Barragan M Metsch L Krawczyk CS Loughlin AM Gardner LI Anderson-Mahoney P Dickinson G del Rio C Antiretroviral T Access Studies Study G Effect of physician specialty on counseling practices and medical referral patterns among physicians caring for disadvantaged human immunodeficiency virus-infected populations Clin Infect Dis 2003 36 1577 1584 10.1086/375070 12802759

[B15] Sheth AN Moore RD Gebo KA Provision of general and HIV-specific health maintenance in middle aged and older patients in an urban HIV clinic AIDS Patient Care STDS 2006 20 318 325 10.1089/apc.2006.20.318 16706706

[B16] Reinhold JP Moon M Tenner CT Poles MA Bini EJ Colorectal cancer screening in HIV-infected patients 50 years of age and older: missed opportunities for prevention Am J Gastroenterol 2005 100 1805 1812 10.1111/j.1572-0241.2005.50038.x 16086718

[B17] Leece P Kendall C Touchie C Pottie K Angel JB Jaffey J Cervical cancer screening among HIV-positive women. Retrospective cohort study from a tertiary care HIV clinic Can Fam Physician 2010 56 e425 e431 21375064 PMC3001950

[B18] Chu C Selwyn PA An epidemic in evolution: the need for new models of HIV care in the chronic disease era J Urban Heal 2011 88 556 566 10.1007/s11524-011-9552-y PMC3126936 21360244

[B19] Iron K Lu H Manuel D Henry D Gershon A Using linked health administrative data to assess the clinical and healthcare system impact of chronic diseases in Ontario Healthc Q 2011 14 23 27 10.12927/hcq.2011.22486 21841373

[B20] Antoniou T Zagorski B Loutfy MR Strike C Glazier RH Validation of case-finding algorithms derived from administrative data for identifying adults living with human immunodeficiency virus infection PLoS One 2011 6 e21748 10.1371/journal.pone.0021748 21738786 PMC3128093

[B21] Matheson FI Dunn JR Smith KLW Moineddin R Glazier RH Canadian marginalization index user guide 2012 Toronto, ON: Canadian Journal of Public Health 10.1007/BF03403823 PMC6973681 23618065

[B22] Kralj B Measuring “rurality” for purposes of health care planning: an empirical measure for Ontario Ont Med Rev 2000 67 33 52

[B23] Gershon AS Wang C Guan J Vasilevska-Ristovska J Cicutto L To T Identifying patients with physician-diagnosed asthma in health administrative databases Can Respir J 2009 16 183 188 20011725 10.1155/2009/963098 PMC2807792

[B24] Tu K Campbell NR Chen Z-L Cauch-Dudek KJ McAlister FA Accuracy of administrative databases in identifying patients with hypertension Open Med 2007 1 e18 e26 20101286 PMC2801913

[B25] Tu JV Naylor CD Austin P Temporal changes in the outcomes of acute myocardial infarction in Ontario, 1992–1996 CMAJ 1999 161 1257 1261 10584086 PMC1230787

[B26] Hux JE Ivis F Flintoft V Bica A Diabetes in Ontario: determination of prevalence and incidence using a validated administrative data algorithm Diabetes Care 2002 25 512 516 10.2337/diacare.25.3.512 11874939

[B27] Steele LS Glazier RH Lin E Evans M Using administrative data to measure ambulatory mental health service provision in primary care Med Care 2004 42 960 965 10.1097/00005650-200410000-00004 15377928

[B28] Huntley AL Johnson R Purdy S Valderas JM Salisbury C Measures of multimorbidity and morbidity burden for use in primary care and community settings: a systematic review and guide Ann Fam Med 2012 10 134 141 10.1370/afm.1363 22412005 PMC3315139

[B29] Canadian Institue for Health Information Making sense of health indicators: statisical considerations 2010 Ottawa, ON: CIHI

[B30] Breslow NE Day NE Statistical methods in cancer research, volume II: the design and analysis of cohort studies 1987 New York: Oxford University Press 61 64 3329634

[B31] Butt AA McGinnis K Rodriguez-Barradas MC Crystal S Simberkoff M Goetz MB Leaf D Justice AC HIV infection and the risk of diabetes mellitus AIDS 2009 23 1227 1234 10.1097/QAD.0b013e32832bd7af 19444074 PMC2752953

[B32] Crothers K Butt AA Gibert CL Rodriguez-Barradas MC Crystal S Justice AC Increased COPD among HIV-positive compared to HIV-negative veterans Chest 2006 130 1326 1333 10.1378/chest.130.5.1326 17099007

[B33] Goulet JL Fultz SL Rimland D Butt A Gibert C Rodriguez-Barradas M Bryant K Justice AC Aging and infectious diseases: do patterns of comorbidity vary by HIV status, age, and HIV severity? Clin Infect Dis 2007 45 1593 1601 10.1086/523577 18190322 PMC3687553

[B34] Salter ML Lau B Go VF Mehta SH Kirk GD HIV infection, immune suppression, and uncontrolled viremia are associated with increased multimorbidity among aging injection drug users Clin Infect Dis 2011 53 1256 1264 10.1093/cid/cir673 21976463 PMC3214585

[B35] Röling J Schmid H Fischereder M Draenert R Goebel FD HIV-associated renal diseases and highly active antiretroviral therapy-induced nephropathy Clin Infect Dis 2006 42 1488 1495 10.1086/503566 16619164

[B36] Vance DE Mugavero M Willig J Raper JL Saag MS Aging with HIV: a cross-sectional study of comorbidity prevalence and clinical characteristics across decades of life J Assoc Nurses AIDS Care 2011 22 17 25 10.1016/j.jana.2010.04.002 20471864

[B37] Barnett K Mercer SW Norbury M Watt G Wyke S Guthrie B Epidemiology of multimorbidity and implications for health care, research, and medical education: a cross-sectional study Lancet 2012 380 37 43 10.1016/S0140-6736(12)60240-2 22579043

[B38] Broemeling A Watson D Black C Chronic conditions and co-morbidity among residents of British Columbia 2005 Vancouver, BC, Canada: Centre for Health Services and Policy Research

[B39] Agborsangaya CB Lau D Lahtinen M Cooke T Johnson JA Multimorbidity prevalence and patterns across socioeconomic determinants: a cross-sectional survey BMC Public Health 2012 12 201 10.1186/1471-2458-12-201 22429338 PMC3353224

[B40] Marengoni A Winbald B Karp A Fratiglioni L Prevalence of chronic diseases and multimorbidity among the elderly population in Sweden Am J Public Heal 2008 98 1198 1200 10.2105/AJPH.2007.121137 PMC2424077 18511722

[B41] Public Health Agency of Canada HIV/AIDS epi update: national HIV prevalence and incidence estimates in Canada for 2008 2010 Ottawa, Ontario, Canada: Surveillance and Risk Assessment Division, Centre for Communicable Diseases and Infection Control

[B42] Glazier RH Zagorski BM Rayner J Comparison of primary care models in Ontario by demographics, case mix and emergency department use, 2008/09 to 2009/10. ICES investigative report 2012 Toronto, Ontario: ICES Investigative Report. Toronto: Institute for Clinical Evaluative Sciences; 2012

[B43] Justice AC Braithwaite RS Lessons learned from the first wave of aging with HIV AIDS 2012 26 Suppl 1 S11 S18 December 2011 22781174 10.1097/QAD.0b013e3283558500 PMC5596448

[B44] Monroe AK Chander G Moore RD Control of medical comorbidities in individuals with HIV J Acquir Immune Defic Syndr 2011 58 458 462 10.1097/QAI.0b013e31823801c4 22083037 PMC3225195

[B45] Aberg JA Gallant JE Ghanem KG Emmanuel P Zingman BS Horberg MA Primary care guidelines for the management of persons infected with HIV: 2013 update by the HIV medicine association of the infectious diseases society of America Clin Infect Dis 2014 58 1 e1 e34 10.1093/cid/cit665 24235263

[B46] Salisbury C Multimorbidity: redesigning health care for people who use it Lancet 2012 380 7 9 10.1016/S0140-6736(12)60482-6 22579042

